# Exploring the Influence of Video Games on Self-Reported Spatial Abilities Among University Students

**DOI:** 10.3390/brainsci14121269

**Published:** 2024-12-17

**Authors:** Reem M. AlWhaibi, Afnan M. Aldhowayan, Shahad M. Alshahrani, Bayan S. Almadi, Reham A. Alamer, Fai M. Albaqami, Eman M. Mortada

**Affiliations:** 1 Department of Rehabilitation Sciences, College of Health and Rehabilitation Sciences, Princess Nourah Bint Abdulrahman University, P.O. Box 84428, Riyadh 11671, Saudi Arabia; rmalwhaibi@pnu.edu.sa (R.M.A.); afnanaldowayan@gmail.com (A.M.A.); shahoody4570@gmail.com (S.M.A.); bayansaudalmadi@gmail.com (B.S.A.); rehamasd93@gmail.com (R.A.A.); 2 Department of Health Communication Sciences, College of Health and Rehabilitation Sciences, Princess Nourah Bint Abdulrahman University, P.O. Box 84428, Riyadh 11671, Saudi Arabia; fay11ff22ff@gmail.com; 3 Family and Community Medicine Department, College of Medicine, Princess Nourah Bint Abdulrahman University, P.O. Box 84428, Riyadh 11671, Saudi Arabia

**Keywords:** video games, spatial abilities, game genres, gender differences, university students

## Abstract

**Background:** Video games are no longer just entertainment; they are increasingly recognized for their potential to enhance cognitive abilities, including spatial cognition. This skill is vital in academic disciplines, where strong spatial reasoning is essential for problem-solving and success. **Aims:** This study investigates how video game engagement impacts self-reported spatial abilities in university students, focusing on the frequency, types, and duration of gaming. It also explores the contributions of specific video game genres and features to perceived cognitive improvements. **Method:** A cross-sectional study was conducted with 566 Saudi university students who completed an online questionnaire on their gaming habits and self-reported spatial abilities. Data were analyzed using independent sample *t*-tests and chi-square tests to assess the associations between video game behaviors and self-reported spatial cognition. **Results:** Frequent gamers (65% of participants) demonstrated significantly higher self-reported spatial abilities than infrequent gamers, particularly in adapting to spatial challenges (*p* < 0.001). Players engaged with action and open-world games reported the greatest perceived improvements in spatial cognition. No significant gender differences were observed, indicating that both males and females benefit equally from gaming. The use of perspective in games was notably linked to spatial skill enhancement (*p* = 0.05). **Conclusions:** Regular video game play, especially with spatially demanding genres, is associated with significant self-reported improvements in spatial abilities. These findings highlight the potential of video games as tools for enhancing self-reported spatial cognition in education and professional training, particularly in STEM fields.

## 1. Introduction

Video games (VGs) have undergone a significant transformation over the past few decades, evolving from simple forms of entertainment to complex, immersive platforms that engage users both cognitively and physically. With advancements in technology and the proliferation of devices such as smartphones, tablets, and gaming consoles, video games have become a major part of daily life for billions of people worldwide. As of 2024, there are approximately 3.09 billion gamers globally, spanning various demographics and age groups [1]. In Saudi Arabia alone, the prevalence of gaming enthusiasts has reached 67% of the population, representing over 23.5 million individuals, according to the National Gaming Esports Strategy [2]. This widespread participation in gaming has sparked interest among researchers in understanding how such digital platforms affect cognitive and behavioral functions.

Traditionally, video games have been viewed as a recreational activity, providing entertainment through interactive digital environments. However, over time, researchers have recognized the potential of video games to influence cognitive abilities, particularly those that are essential for academic success and everyday functioning [3]. Video games are no longer confined to entertainment; they have increasingly been used as tools for enhancing learning, problem-solving, and other higher-order cognitive skills. Players must navigate through complex levels, solve intricate puzzles, and often engage in competitive environments that require quick thinking and adaptability [4]. As such, video games provide a rich environment for studying cognitive processes, including spatial awareness (SA), attention, memory, and executive functioning [5,6].

One of the most interesting aspects of video gaming is its impact on spatial cognition, which includes skills such as spatial awareness, orientation, and navigation. SA refers to the ability to understand, reason, and remember the spatial relationships between objects, which is crucial for tasks like moving through a physical environment, manipulating objects, or understanding visual and spatial data [7]. For university students, spatial abilities are critical not only for academic tasks, such as interpreting graphs, diagrams, and models in subjects like mathematics and physics, but also for practical activities such as navigating campus or organizing study materials effectively [8]. The dynamic and interactive nature of video games makes them an ideal platform for enhancing these skills, as they often require players to make quick decisions based on their understanding of space and spatial relationships [9].

Research into the cognitive effects of video games has yielded promising results, particularly concerning the enhancement of spatial cognition. Studies have shown that action video games, which often involve navigating through complex virtual environments, can lead to improvements in spatial resolution, attention, and visual-motor coordination [6,10]. Players who frequently engage with video games that challenge their spatial abilities, such as action, puzzle, and strategy games, often demonstrate superior performance in spatial tasks compared to non-gamers [11,12]. This is because video games offer a unique combination of visual, auditory, and physical stimuli that can enhance the brain’s ability to process spatial information [13].

The relationship between video games and cognitive development has been studied across a variety of contexts, with researchers focusing on how video games affect memory, attention, and problem-solving abilities [14]. In particular, action video games have been found to improve attentional control and working memory, both of which are closely linked to spatial cognition [6,15]. Video games have been shown to improve multitasking abilities and attention spans, which are critical for navigating complex virtual environments and completing spatial tasks [3]. Furthermore, the physical feedback provided by some video games, such as muscle strain from in-game actions, can contribute to a player’s spatial experience by simulating real-world physical interactions [13]. These findings suggest that video games have the potential to significantly enhance spatial awareness, a skill that is essential for both academic and real-world problem-solving.

In academia, spatial awareness plays a crucial role in learning and performance, particularly in STEM (science, technology, engineering, and mathematics) disciplines [16]. Research has consistently shown that individuals with strong spatial abilities are more likely to excel in STEM-related fields, as these disciplines require a deep understanding of spatial relationships, visual–spatial reasoning, and the ability to manipulate abstract concepts [17]. In fact, spatial skills have been identified as one of the strongest predictors of success in mathematics and science, making them a key focus of cognitive training programs aimed at enhancing STEM performance [18]. For university students, the development of spatial abilities is particularly important, as it influences their ability to comprehend complex diagrams, solve spatially demanding problems, and navigate academic challenges [8]. As educational institutions continue to explore innovative ways to enhance student learning, video games are increasingly being recognized as valuable tools for developing spatial cognition and other cognitive skills [19].

One prominent example of the educational potential of video games is the use of *Minecraft*, a sandbox game that allows players to build and explore virtual worlds. *Minecraft* has been widely adopted in educational settings to promote creativity, problem-solving, and spatial reasoning among students [20,21]. The game’s open-ended nature encourages critical thinking and spatial exploration, as players must manipulate blocks to construct complex structures and navigate virtual landscapes. Research by Rosas and Nussbaum [22] found that students who engaged with educational video games exhibited significant improvements in cognitive skills such as problem-solving, strategic thinking, and spatial awareness. Similarly, Gee [19] argues that video games can enhance players’ literacy skills, including spatial literacy, by challenging them to navigate and interpret complex virtual environments. The adaptability of video games, which allows for personalized learning experiences, makes them particularly effective for developing cognitive skills such as attention, memory, and decision-making [6,14].

While the benefits of video games on cognitive development are well-documented, there remains a noticeable gap in the literature regarding their specific impact on spatial abilities, particularly among university students. Although numerous studies have explored the effects of video games on general cognitive functions such as memory, attention, and decision-making, fewer studies have focused on how video game engagement influences spatial cognition in higher education settings [5,14]. This gap is particularly important to address, as university students are frequently exposed to video games and are often required to engage with spatially demanding academic tasks. Furthermore, while some studies have examined the relationship between video games and spatial awareness, the majority have focused on broader cognitive domains, leaving the specific influence of video games on spatial abilities relatively underexplored [3,9].

This study seeks to address this gap by exploring the influence of video games on self-reported spatial abilities among Saudi university students. By examining the frequency, types, and durations of video game engagement, this study aims to provide a clearer understanding of how different gaming habits impact self-reported spatial cognition. Saudi university students represent a population underrepresented in cognitive and gaming research. The cultural, educational, and societal dynamics unique to Saudi Arabia, coupled with its Vision 2030 initiative emphasizing the integration of technology into education, make this group particularly relevant for understanding how gaming influences cognitive skills such as spatial cognition.

Furthermore, this study will investigate potential variations in self-reported spatial abilities between frequent video game players and non-frequent ones, considering specific video game genres or features that may contribute to these differences. This study aims to describe patterns and associations between gaming behaviors and self-reported spatial abilities without inferring causation. By focusing on this population, the study provides culturally specific insights that contribute to the growing body of literature on the cognitive benefits of video games and their potential applications in educational settings, expanding research beyond the predominantly Western contexts explored thus far.

## 2. Method

### 2.1. Study Design and Setting

This cross-sectional study was conducted from January to May 2024 to assess the impact of video game exposure on spatial abilities among university students in Saudi Arabia. The cross-sectional design was chosen because it allows for the efficient collection of data from a large sample within a limited timeframe. This design is particularly suitable for exploratory research aiming to identify associations between video game behaviors and self-reported spatial abilities at a specific point in time.

### 2.2. Ethical Considerations

The study protocol, including the questionnaire and data collection methods, received approval from the Institutional Review Board of Princess Nourah bint Abdulrahman University (approval number: 24-0585, issued on 5 March 2024). Participation in the study was entirely voluntary and anonymous, with no personal information collected. Participants faced no risks, incurred no costs, and received no financial compensation for their involvement. They were free to withdraw from the study at any time without facing any consequences. Informed consent was obtained electronically, with participants confirming their understanding and agreement by selecting “Yes” before starting the questionnaire. These steps ensured the study complied with the highest ethical standards, safeguarding participants’ rights and confidentiality throughout the process.

### 2.3. Population and Sample Size Calculation

According to the Ministry of Education in 2023, the total population of university students in Saudi Arabia is 1,297,426. Using OpenEpi software version 3, the required sample size was calculated with a 95% confidence interval and a *p*-value of ≤0.05, yielding an estimated sample size of 400 (*n* = 385).

### 2.4. Sampling Strategy

**Figure 1:**
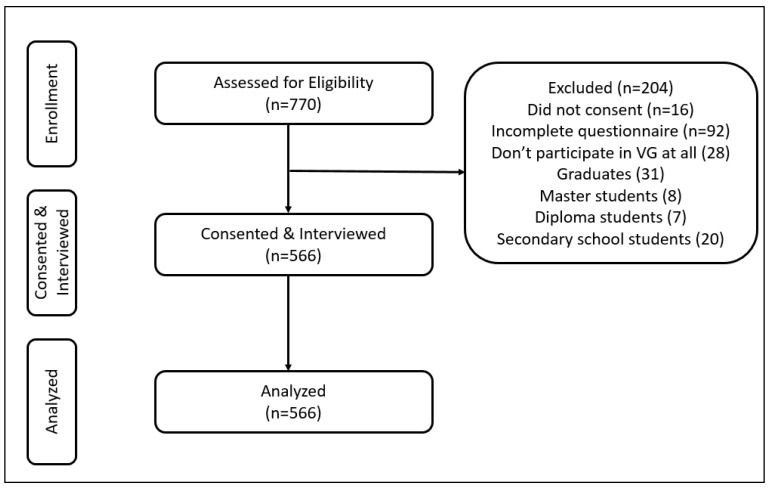
Flowchart of participants’ recruitment.

A convenience sampling technique was employed to facilitate participant recruitment within a defined timeframe. A total of 566 participants were initially recruited and refined according to the following inclusion criteria: Saudi undergraduate university students aged 17 to 25, of any gender, who self-identified as video gamers and had been playing video games for at least two years. Figure 1 demonstrates the stages of recruitment, inclusion and exclusion criteria, and the final sample size used for analysis.

### 2.5. Measuring Tool and Its Development

The questionnaire used in this study was designed to explore the relationship between video game engagement and spatial abilities among Saudi university students. It focused on capturing key information regarding the participants’ gaming habits, cognitive skills, and spatial awareness. The questionnaire comprised four sections and a total of 41 questions, structured to gather comprehensive data on the participants’ demographics, video game behaviors, and self-reported spatial abilities.

The first section of the questionnaire served as an introduction and informed consent, ensuring that participants were fully aware of the study’s aims, procedures, and their rights. This section confirmed voluntary participation, anonymity, and withdrawal rights. Participants could only proceed with the survey after providing electronic consent by selecting “Yes” to acknowledge their understanding of the study details.

The second section gathered demographic information, including age, gender, academic major, academic level, GPA, and family income. The third section was focused on video game behaviors and included questions about the frequency, duration, and types of video games played, as well as the devices used. Participants were asked to rank their preferred video game genres and specify their gaming habits, such as whether they played solo or in multiplayer settings and whether they played sitting or actively. The fourth section aimed to assess participants’ spatial abilities in relation to their gaming behaviors. To ensure clarity, an explanation of spatial ability was provided before introducing the questions. The participants were asked to self-assess their spatial abilities and indicate whether they believed video games had improved these abilities. This section included Likert scale questions on the perceived impact of specific video game genres and features, such as 3D environments, puzzles, and map navigation, on spatial cognition. Additionally, participants were asked to reflect on whether their video gaming experiences translated into improved spatial awareness in real-world contexts, including their navigation skills, attention to detail, and memory.

The questionnaire was designed to be completed within approximately 10 min, and it included a combination of multiple-choice and Likert scale questions. No identifying information was collected, ensuring participant confidentiality. The questionnaire was administered online using Google Forms, and participation was voluntary. Responses were used solely for research purposes, and the data were analyzed in aggregate to explore patterns and trends among the sample.

### 2.6. Pilot Testing and Validation

Before the full-scale implementation of the questionnaire, a thorough pilot testing and validation process was conducted. This phase included feedback from two subject matter experts, along with trial runs involving 27 students. The goal was to evaluate the clarity, comprehension, and relevance of the questionnaire. Based on the feedback, adjustments were made to improve its effectiveness and ensure the tool’s validity.

To assess the reliability of the questionnaire, Cronbach’s alpha was calculated for the items related to self-reported spatial abilities. The resulting Cronbach’s alpha value was 0.87, indicating good internal consistency. Construct validity was evaluated through expert feedback, ensuring that the items were representative of the intended constructs. Refinements to question phrasing and content were implemented based on this feedback to enhance alignment with the study’s objectives. These steps ensured the questionnaire was both reliable and valid for the study population.

### 2.7. Data Collection

The finalized Arabic version of the questionnaire was distributed electronically via Google Forms to students at Saudi universities. Participants were provided with detailed instructions outlining the study’s purpose, confidentiality assurances, and the voluntary nature of their participation. Informed consent was obtained electronically before participants could proceed with the questionnaire, ensuring they fully understood the study’s objectives and their rights. The questionnaire could be completed at participants’ convenience, and all responses were anonymized to safeguard confidentiality and privacy. The data collection period spanned ten weeks, from March to May 2024, providing ample time to gather responses. Once data collection was concluded, the responses were compiled and underwent comprehensive statistical analysis using SPSS software (version 28).

### 2.8. Statistical Analysis

The data collected from the study were analyzed using the Statistical Package for Social Sciences (SPSS) version 23. Descriptive statistics, including means, standard deviations, and frequencies, were used to summarize the demographic characteristics and gaming behaviors of the participants. The chi-square test was employed to examine the association between video game exposure (frequent vs. infrequent) and personal characteristics, such as age, gender, academic major, and academic year.

By analyzing the relationship between video game exposure and spatial abilities, independent sample *t*-tests were conducted to compare the mean scores of spatial abilities and cognitive skills between frequent and infrequent video game players. The *t*-tests were also used to assess differences in specific features of video games, such as complex spatial orientation challenges and navigation skills, and their impact on spatial abilities in real-world contexts. Additionally, a Student’s *t*-test was chosen based on assessing the normality using a Kolmogorov–Smirnov test to evaluate the mean differences between groups on their perceptions of how video games influence their spatial abilities, cognitive skills, and adaptation to spatial challenges in virtual environments. The effect size of statistically significant differences was measured by Cohen’s *d*. Overall, statistical significance was defined as *p* ≤ 0.05.

## 3. Results

A total sample size of 770 individuals, with 566 valid responses (73%) included in the study. A total of 204 participants were excluded due to various reasons, including refusal to participate, lack of video game experience, being graduates, master’s, diploma, or secondary school students, or providing incomplete or incorrect information (Figure 1).

According to Table 1, the participants were categorized according to their frequency of exposure to VGs into two groups: 368 students categorized as frequently exposed to VGs, with a prevalence of 65.0%, and 198 students categorized as infrequently exposed to VGs, accounting for 35.0%. Personal characteristics of the study groups are illustrated in Table 1, with the overall mean age (20.25 ± 1.58); about 62% were in the age group ≤ 20 years old, and most of them (65.4%) were females. The percentage of students from humanities colleges was 44.3%, and from sciences colleges was 29.9%. Most of the students (86.4%) had a family’s monthly income of more than 5000 SAR.

Moreover, a chi-square test was used to measure the relationship between the frequency of VGs exposure and personal characteristics of the participants. The only significant association was with the students’ academic year, whereas VG exposure was significantly higher among students enrolled in the fourth year (*p* = 0.04). No other significant association existed between both study groups and the remaining personal characteristics (Ps > 0.05) (Table 1).

In terms of the relationship between VGs exposure and associated behaviors among the participants, a significant association was detected between both study groups and how often they play video games and how long they spend playing in a single session, with the majority of students frequently exposed to VGs play daily and >3 h (96.3% and 83.5%, *p* < 0.001). Likewise, a significant association was found with how they usually play, with most students frequently exposed to VGs play actively (86.2%, *p* = 0.009) (Table 2).

Table 3 shows the relationship between VGs exposure and how it influences their overall assessment of their SAs. A significant association was found between both study groups, and that video games can enhance spatial abilities with most students frequently exposed to VGs strongly agreeing that VGs can enhance spatial abilities (74.2%, *p* < 0.001), with a high frequency rate of their overall spatial abilities as very good (71.8%, *p* < 0.001). Another significant association was found with how well they can adapt to new spatial challenges and remain aware of in-game elements while playing video games in changing environments (*p* < 0.001).

On assessing the relationship between VGs exposure and the extent to which the participants believe that playing VGs can improve their SAs, the Student *t*-test revealed that the mean score for the participants frequently exposed to VGs was significantly higher than the infrequently exposed in terms of the improvement in SAs because of playing VGs (3.7 ± 1.02 vs. 3.9 ± 0.94, t (565) = −3.68, *p* < 0.001). The difference between the study groups resulted in a large effect size (d = 0.98). The mean score for the participants frequently exposed to VGs was significantly higher than the infrequently exposed in terms of the improvement in their cognitive skills because of playing VGs (3.81 ± 1.13 vs. 4.13 ± 1.06, t (565) = −3.39, *p* = 0.001). The difference between likely and unlikely groups resulted in a small effect size (d = 0.29).

Likewise, the mean scores for the participants who were frequently exposed to VGs were significantly higher than those who were infrequently exposed to VGs with complex spatial orientation challenges, and mastering navigation skills in VGs leads to improved navigation abilities in real-world situations (3.9 ± 1.05 vs. 4.14 ± 0.97, t (565) = −2.29, *p* =.02, and 3.92 ± 1.02 vs. 4.09 ± 0.93, t (565) = −2.11, *p* = 0.04, respectively). In addition, the mean score of the storyline and character development in VGs affects SA development, and practicing SA in VGs can positively affect spatial awareness in the real world. The results were higher among the students frequently exposed to VGs (3.88 ± 1.02 vs. 4.07 ± 0.97, t (565) = −2.19, 3.76 ± 1.07 vs. 3.96 ± 1.02, t (565) = −2.14, and *p* = 0.03, respectively) (Table 4).

On assessing the relationship between VGs exposure and participant opinion about specific features, in-game activities, and visual cues of video games that contribute most to developing spatial skills (Table 5). Only the perspectives of the visual cues showed a significant association (*p* = 0.05), while all remaining variables of the visual cues, in-game activities, and specific features showed no significant association (Ps > 0.05).

## 4. Discussion

The study aimed to explore the relationship between video game exposure and spatial abilities among university students, examining the frequency, types, and duration of video game engagement. According to the findings, 65% of the participants were categorized as frequent video game players, with a significant portion (71.8%) rating their spatial abilities as “very good”. The results clearly demonstrate that frequent exposure to video games significantly enhances self-reported spatial awareness, which aligns with previous research showing that video games can improve spatial reasoning, navigation skills, and cognitive flexibility [5,16].

In particular, the frequent video gamers reported significantly higher abilities in adapting to new spatial challenges and remaining aware of in-game elements in dynamic environments as compared to infrequent gamers. The study showed significant differences between frequent and infrequent gamers in their perception of spatial abilities. This supports the hypothesis that video game exposure positively influences spatial cognition, likely due to the nature of many games that require players to navigate complex environments and process spatial information rapidly.

### 4.1. Video Game Exposure and Spatial Abilities

One of the central findings of this study is the strong association between frequent video game play and self-reported improvements in spatial abilities. The data showed that students who engaged with video games daily, especially for longer periods, demonstrated significantly higher spatial awareness and adaptation to spatial challenges. These results are consistent with previous research highlighting the cognitive benefits of video games. For instance, Green and Bavelier [5] found that action video games enhance spatial resolution and visual processing, which are key components of spatial awareness. Similarly, Bediou and Adams [23] conducted a meta-analysis revealing that action video games, compared to non-gaming or less spatially demanding games, result in substantial improvements in spatial cognition.

Video games, particularly those requiring navigation through 3D environments, present users with tasks involving spatial reasoning, mental rotation, and object tracking—skills directly related to spatial abilities [24]. This aligns with our finding that students frequently exposed to such games rated their spatial abilities as “very good” more often than those who played less frequently. The repeated interaction with virtual environments in games like *Minecraft*, *Assassin’s Creed*, and *Call of Duty* provides players with ongoing opportunities to refine their spatial skills, which, over time, transfer to real-world situations. This is supported by the work of Uttal and Meadow [16], who argue that spatial cognition is highly malleable and can be improved through targeted practice, including video game play.

### 4.2. Frequency and Duration of Video Game Play

The results also indicated a strong relationship between the frequency and duration of video game play and spatial ability improvements. Participants who played video games daily or for more than three hours per session reported significantly higher spatial awareness and improved cognitive skills. This aligns with the notion that longer engagement with spatially challenging video games provides more extensive opportunities for players to practice and refine their spatial abilities. For instance, students who played video games for more than three hours were significantly more likely to report improvements in their spatial abilities, with 83.5% of frequent gamers reporting such improvements. These findings support the idea that consistent and prolonged exposure to video games can lead to measurable cognitive benefits, particularly in spatial reasoning and navigation [25,26].

### 4.3. Impact of Specific Video Game Genres

The study explored how specific video game features, such as 3D environments, map exploration, and visual cues, contribute to spatial abilities. While most of these features did not show a statistically significant association with improved spatial skills, one possible explanation for this lack of significance is the variability in how participants interpreted the term “puzzle resolution”, which might encompass diverse actions like solving complex problems, rotating objects, or moving pieces. However, one notable exception was the use of perspective, which was found to have a significant association with spatial improvements. This suggests that games that require players to adopt varying perspectives and navigate complex environments may offer unique cognitive benefits related to spatial reasoning.

Supporting this finding, research by Dale and Joessel [24] demonstrated that games involving perspective shifts, such as first-person shooters (*Call of Duty*) or third-person adventure games (*The Legend of Zelda: Breath of the Wild*), help players improve their ability to mentally manipulate objects and navigate through space. These types of games often require players to adapt quickly to changing viewpoints, enhancing their spatial flexibility and perceptual abilities.

Additionally, while features such as shadows, lighting, and depth of field did not show significant associations in this study, this does not completely discount their role in enhancing spatial immersion. Games that incorporate realistic visual cues might offer subtle improvements in spatial cognition by providing a more immersive experience, even if these features alone do not lead to measurable improvements. For example, open-world exploration games like *Minecraft* often use such visual elements to create a more authentic spatial environment, but the benefits of these features might only manifest in combination with more interactive game mechanics like navigation and puzzle-solving.

In this study, games like *Minecraft* and *Call of Duty* were commonly cited by participants as contributing to improvements in their spatial abilities, aligning with the significant association between perspective and spatial skills. Games that challenge players to navigate large, open-world environments and solve spatial puzzles are particularly beneficial in enhancing spatial reasoning, as they require ongoing engagement with spatially dynamic tasks [25,26].

### 4.4. Real-World Applications of Improved Spatial Abilities

The findings of this study provide compelling evidence that video games can serve as effective cognitive training tools, particularly in educational and professional settings where spatial abilities are critical. The significant improvements in spatial abilities observed among frequent gamers suggest that video games could be used to enhance spatial reasoning, which is essential for success in many STEM (science, technology, engineering, and mathematics) fields. Research by Uttal and Meadow [16] has highlighted the strong link between spatial cognition and proficiency in disciplines such as mathematics, physics, and engineering. This suggests that students with enhanced spatial abilities are more likely to excel in these areas, making video games a potentially valuable tool for academic performance enhancement.

Cultural considerations are particularly relevant in the Saudi context, where gaming is rapidly gaining popularity, with over 67% of the population engaging in video gaming activities. However, societal attitudes toward gaming and its role in education are evolving. Historically, video gaming in Saudi Arabia has been perceived primarily as a form of entertainment, but increasing awareness of its cognitive benefits offers opportunities for integration into educational frameworks. For example, gamified learning platforms and educational games are gaining traction in schools and universities, aligning with Saudi Arabia’s Vision 2030 goals to incorporate technology into education. Highlighting these cultural shifts provides context for the significance of this study’s findings and underscores the potential for video games to bridge traditional teaching methods with innovative digital tools.

Globally, video games have been successfully integrated into educational programs to enhance spatial reasoning and other cognitive skills. For instance, games like Minecraft are used to teach geometry and spatial concepts, while Portal 2 is employed to develop problem-solving skills. In Saudi Arabia, introducing similar gamified tools in STEM education could align with national efforts to foster creativity and innovation among students. Additionally, cultural and regional adaptations of these programs could address the specific educational needs of Saudi students, making video games a more accessible and impactful tool for learning.

The data from this study demonstrated that frequent video gamers rated their ability to adapt to new spatial challenges in real-world environments significantly higher than infrequent gamers. This finding underscores the potential of video games to provide transferable skills that extend beyond virtual environments. The ability to mentally manipulate objects, navigate through space, and solve spatial problems in gaming contexts translates into real-world benefits, which can be particularly useful in fields requiring quick decision-making and spatial awareness, such as aviation, surgery, and military operations [27,28,29]. Simulation games, in particular, are already used in these professional domains to train individuals in environments where spatial reasoning and rapid, precise judgments are critical.

Furthermore, this study found that open-world games were especially effective in fostering spatial skills. These games, which often require players to explore large, complex environments and adapt to spatial challenges, provide a highly immersive experience that mirrors real-world navigation tasks. As supported by prior research, the cognitive benefits from these games can be utilized in professional training programs where navigation and spatial orientation are important [23]. For example, pilots, surgeons, and military personnel may benefit from video game-based training that enhances their ability to process spatial information rapidly and effectively.

In educational settings, video games that emphasize spatial reasoning can be incorporated into curricula to engage students in a more interactive and effective learning experience. By using video games as educational tools, instructors can help students develop spatial skills that are fundamental to understanding abstract concepts in subjects like geometry, physics, and engineering. The integration of video games into education could help bridge gaps in traditional teaching methods by offering an engaging, hands-on approach to learning spatially demanding subjects [19].

Moreover, video games have potential applications in rehabilitation programs for individuals with spatial impairments. Interactive games that involve navigating virtual environments or solving spatial puzzles can provide a motivating and enjoyable way for patients to improve their spatial cognition in a structured setting. This is particularly relevant for populations recovering from strokes or brain injuries, where spatial awareness and coordination may be impaired [30,31,32,33].

### 4.5. Implications of Cross-Sectional Design on Results

The cross-sectional nature of this study provides valuable insight into the associations between video game exposure and self-reported spatial abilities. However, this design limits the ability to infer causation, as the data represent only a snapshot of the participants’ experiences at a single point in time. For example, it remains unclear whether video gaming directly improves spatial abilities or whether individuals with inherently better spatial skills are more inclined to play video games.

### 4.6. Comparison with Previous Studies

The findings of this study align with prior research emphasizing the positive impact of video gaming on spatial cognition. For instance, [5] reported significant enhancements in spatial resolution and visual processing among frequent action video game players. Similarly, Uttal and Meadow [16] demonstrated that targeted video game practice improves spatial reasoning, navigation skills, and cognitive flexibility. Our findings corroborate these studies by showing that frequent gamers report higher spatial abilities, particularly in adapting to spatial challenges and maintaining situational awareness in dynamic environments.

However, this study also diverges from prior research in some respects. While studies like Bediou and Adams [23] and Martinez and Gimenes [12] emphasized gender differences in cognitive improvements from gaming, this study found no significant gender-based disparities, suggesting that video gaming benefits spatial cognition equally across genders. Additionally, whereas prior studies such as Dale and Joessel [24] highlighted the role of immersive game features like 3D environments and dynamic terrains in improving spatial skills, our findings indicate that perspective-related features hold a more significant association with spatial cognition in this population.

The specific focus on Saudi university students provides novel insights into cultural and regional gaming behaviors, contributing to the growing body of evidence on the cognitive benefits of video games in diverse populations. These findings highlight the potential for educational and professional applications of video gaming in contexts such as STEM education and skill development, as previously suggested by researchers like Gee [19] and Rosas and Nussbaum [22].

## 5. Conclusions

The study reveals that frequent video gaming significantly improves spatial cognition, especially with action and open-world games. These findings support the integration of video games into educational curricula and professional training programs to enhance spatial reasoning, which is crucial for success in STEM fields, aviation, surgery, and other professions. Gender-based differences in spatial ability improvements were not significant, indicating that video games offer cognitive benefits to both male and female players.

To integrate video games into educational curricula effectively, it is essential to consider both technological and pedagogical factors. For instance, schools and universities could adopt gamified learning platforms or use commercially available games with proven cognitive benefits, such as Minecraft for spatial reasoning or Portal 2 for problem-solving skills [34,35]. However, successful integration would require adequate infrastructure, such as access to gaming devices and internet connectivity, as well as teacher training to align game-based activities with learning objectives. Pedagogically, video games should be used as supplementary tools rather than standalone methods, incorporated into a blended learning approach where students engage with traditional and digital resources. Clear guidelines for selecting games based on their cognitive impact and alignment with curriculum goals are also necessary to maximize their educational potential.

### Limitations and Future Research

While the findings of this study provide valuable insights into the cognitive benefits of video game exposure, several limitations should be acknowledged. First, the cross-sectional design of the study limits the ability to infer causality, and the observed associations may be influenced by unmeasured confounding variables, such as prior gaming experience or other digital media use. Future longitudinal research should explore the temporal dynamics of video game-related cognitive improvements, assessing whether the effects are sustained or temporary.

Additionally, the reliance on self-reported data may introduce bias, as participants may overestimate or underestimate their spatial abilities or gaming habits. Future studies should incorporate objective measures such as psychometric tests, including mental rotation tasks or spatial memory tests, to provide a more accurate assessment of the cognitive impact of video games. Another limitation concerns the use of generalized terms in the questionnaire, such as “puzzle resolution”, which could have been interpreted differently by participants based on their gaming experiences. While the questionnaire was validated and pilot-tested, providing specific examples in future studies—such as “rotating pieces to fit a pattern”—may help reduce ambiguity and improve data consistency.

Finally, while this study focused on Saudi university students, the findings may not be generalizable to other populations. Cross-cultural research is needed to explore whether the cognitive benefits of video games extend to students in different educational systems and cultural contexts.

## Figures and Tables

**Table 1:** Relationship between video game exposure and personal characteristics of the respondent university students (*n* = 566).

Characteristic	Responses	Overall N. (%)	VG Exposure		$\chi^{2}$ Test	*p* Value
			Infrequent N. (%)	Frequent N. (%)		
Age groups (y)	≤20	349 (61.7)	126 (36.1)	223 (63.9)	1.97	0.53
	>20	217 (38.3)	72 (33.2)	145 (66.8)		
	X ± SD	20.23 ± 1.58				
Gender	Male	196 (34.6)	61 (31.1)	135 (68.9)	1.94	0.09
	Female	370 (65.4)	137 (37.0)	233 (63.0)		
Academic year	1st	212 (37.5)	68 (32.1)	144 (67.9)	7.82	0.04 *.
	2nd	146 (25.8)	58 (39.7)	88 (60.3)		
	3rd	106 (18.7)	46 (43.4)	60 (56.6)		
	≥4th	102 (18.0)	26 (25.5)	76 (74.5)		
Academic major (college)	Health Science	115 (20.3)	48 (41.7)	67 (58.3)	5.06	0.17
	Humanities	249 (43.9)	89 (35.7)	160 (64.3)		
	Sciences	169 (29.9)	49 (29.0)	120 (71.0)		
	Language institutes	33 (5.8)	12 (36.4)	21 (63.6)		
Current GPA	<4	216 (38.2)	79 (36.6)	137 (63.4)	0.98	0.29
	≥4	350 (61.8)	119 (34.0)	231 (66.0)		
	X ± SD	3.94 ± 0.97				
Family’s monthly income (SAR)	<5000	77 (13,6)	28 (36.4)	49 (63.6)	1.07	0.44
	≥5000	489 (86.4)	170 (34.8)	319 (65.2)		
Total		566 (100.0)	198 (35.0)	368 (65.0)		

* *p*-value is statistically significant ≤ 0.05, VG: video game, GPA: Grade Point Achievement, SAR: Saudi Riyals.

**Table 2:** Relationship between video game exposure and associated behaviors among the participants (*n* = 566).

Characteristics	Responses	Overall N. (%)	VG Exposure		$\chi^{2}$ Test	*p* Value
			Infrequent N. (%)	Frequent N. (%)		
When did you start playing video games?	<3 years	50 (8.8)	15 (30.0)	35 (70.0)	4.92	0.08
	3–5 years	117 (20.7)	32 (27.4)	85 (72.6)		
	>5 years	399 (70.5)	151 (37.8)	248 (62.2)		
How often do you play video games? **	Three times or less monthly	80 (14.1)	71 (88.7)	9 (11.3)	2.7	<0.000 *
	1/week	36 (6.6)	31 (86.1)	5 (13.9)		
	2/week	54 (9.5)	36 (66.7)	18 (33.3)		
	3/week	56 (9.9)	22 (39.3)	34 (60.7)		
	4/week	80 (14.1)	18 (22.5)	62 (77.5)		
	1/2 days	65 (11.5)	9 (13.8)	56 (86.2)		
	Daily	190 (33.6)	7 (3.7)	183 (96.3)		
	Others (monthly)	5 (0.9)	4 (80.0)	1 (20.0)		
How long do you spend playing in a single session?	<30 min	40 (7.1)	29 (72.5)	11 (27.5)	75.3	<0.000 *
	30–60 min	127 (22.4)	70 (55.1)	57 (44.9)		
	1–2 h	141 (25.4)	45 (31.9)	96 (68.1)		
	2–3 h	137 (24.2)	34 (24.8)	103 (75.2)		
	>3 h	121 (21.4)	20 (16.5)	101 (83.5)		
What is your preferred way of playing?	Solo play	87 (15.4)	42 (48.3)	45 (51.7)	8.6	0.01 *
	Multiplayer	71 (12.5)	26 (36.6)	45 (63.4)		
	Both ways	408 (72.1)	130 (31.9)	278 (68.1)		
How do you usually play?	Play sitting	537 (94.9)	194 (36.1)	343 (63.9)	6.7	0.009 *
	Play actively	29 (5.1)	4 (13.8)	25 (86.2)		
Devices used to play						
Mobile phone or tablet	No		58 (33.7)	114 (66.3)	1.74	0.70
	Yes		140 (35.5)	254 (64.5)		
PlayStation	No	229 (52.8)	78 (34.1)	151 (65.9)	1.43	0.72
	Yes	337 (47.2)	120 (35.6)	217 (64.4)		
PC	No	411 (72.6)	151 (36.7)	260 (63.3)	2.03	0.17
	Yes	155 (27.4)	47 (30.3)	108 (69.7)		
Xbox	No	529 (93.5)	189 (35.7)	340 (64.3)	1.97	0.21
	Yes	37 (6.5)	9 (24.3)	28 (75.7)		
Nintendo Switch	No	519 (91.7)	186 (35.8)	333 (64.2)	2.01	0.2
	Yes	47 (8.3)	12 (25.5)	35 (74.5)		
Virtual reality **	No	552 (97.5)	194 (35.1)	358 (64.9)	0.26	0.78
	Yes	14 (2.5)	4 (28.6)	10 (71.4)		
Total		566 (100.0)	198 (35.0)	368 (65.0)		

* *p*-value is statistically significant ≤ 0.05; ** The Fisher exact test is used; VG: video game.

**Table 3:** Relationship between video game exposure and how it influences their overall assessment of their SAs among the participants (*n* = 566).

Characteristics	Responses	Overall N. (%)	VG Exposure		$\chi^{2}$ Test	*p* Value
			Infrequent N. (%)	Frequent N. (%)		
How do you rate your overall spatial abilities? **	Very poor	5 (0.9)	2 (40.0)	3 (60.0)	24.95	<0.000 *
	Poor	17 (3.0)	12 (70.6)	5 (29.4)		
	Average	120 (21.2)	58 (48.3)	62 (51.7)		
	Good	211 (37.3)	66 (31.3)	145 (68.7)		
	Very good	213 (37.6)	60 (28.2)	153 (71.8)		
Video games can enhance spatial abilities **	Strongly disagree	15 (2.7)	7 (46.7)	8 (53.3)	21.97	<0.000 *
	Disagree	29 (5.1)	16 (55.2)	13 (44.8)		
	Neutral	163 (28.8)	74 (45.4)	89 (54.6)		
	Agree	200 (35.3)	60 (30.0)	140 (70.0)		
	Strongly agree	159 (28.1)	41 (25.8)	118 (74.2)		
How do you rate your attention during video game sessions?	Very low	12 (2.1)	3 (25.0)	9 (75.0)	2.29	0.68
	Low	25 (4.4)	9 (36.0)	16 (64.0)		
	Moderate	167 (29.5)	56 (33.5)	111 (66.5)		
	High	216 (38.2)	72 (33.3)	144 (66.7)		
	Very high	146 (25.8)	58 (39.7)	88 (60.3)		
What aspect of narrative complexity do you think contributes most to the development of spatial ability?	Non-linear storylines	85 (15.0)	36 (42.4)	49 (57.8)	6.80	0.08
	Multi-layered plots	80 (14.1)	19 (23.8)	61 (76.2)		
	Deep character development	170 (30.0)	63 (37.1)	107 (62.9)		
	Complex puzzles embedded in the story	231 (40.8)	80 (34.6)	151 (65.4)		
How aware are you of the surrounding objects when playing VG, especially in dynamic and fast-paced situations?	Very unaware	32 (5.7)	11 (34.4)	21 (65.6)	2.02	0.73
	Unaware	110 (19.4)	37 (33.6)	73 (66.4)		
	Neutral	13924.6)	54 (38.8)	85 (61.2)		
	Aware	201 (35.5)	71 (35.3)	130 (64.7)		
	Very aware	84 (14.8)	25 (29.8)	59 (70.2)		
When playing video games in changing environments, how well can you adapt to new spatial challenges and remain aware of in-game elements?	Very poor	17 (3.0)	3 (17.6)	14 (82.4)	31.27	<0.000 *
	Poor	29 (5.1)	7 (24.1)	22 (75.9)		
	Moderate	213 (37.6)	97 (45.5)	116 (54.5)		
	Good	180 (31.8)	67 (37.2)	113 (62.8)		
	Very good	127 (22.1)	23 (18.1)	104 (81.9)		
Total		566 (100.0)	198 (35.0)	368 (65.0)		

* *p*-value is statistically significant ≤ 0.05; ** The Fisher exact test is used; VG: video game.

**Table 4:** Relationship between video game exposure and the extent to which the participants believe that playing VGs can improve their SAs (*n* = 566).

Characteristics	Mean Difference	VG Exposure		T Test	*p* Value	d^t^
		Infrequent M ± SD	Frequent M ± SD			
Noticed an improvement in SAs as a result of playing VGs.	−0.32	3.7 ± 1.02	3.9 ± 0.94	−3.68	0.000 *	−0.20
Spatial elements in VGs are important for developing SAs.	−0.20	3.9 ± 1.1	4.13 ± 0.87	−2.46	0.01 *	−0.23
Noticed a correlation between specific game features and improvement in Sas.	−0.26	4.03 ± 0.98	4.30 ± 0.90	−3.21	0.001 *	0.98
Noticed an improvement in cognitive skills as a result of playing VG.	−0.33	3.81 ± 1.13	4.13 ± 1.06	−3.39	0.001 *	−0.29
Open-world game settings contribute more to the development of spatial skills compared to linear or closed environments.	−0.18	3.8 ± 1.06	4.01 ± 1.06	−1.91	0.06	
Incorporating virtual reality (VR) features in VGs affects the development of spatial skills.	−0.06	3.7 ± 1.15	3.72 ± 1.16	−0.58	0.56	
Playing VGs in open-world settings, it is easy to adapt to spatial orientation within the game.	−0.12	3.81 ± 1.01	3.93 ± 1.06	−1.37	0.18	
VGs with complex spatial orientation challenges improve navigation skills in the real world.	−0.20	3.9 ± 1.05	4.14 ± 0.97	−2.29	0.02 *	−0.24
Practicing SA in VGs can positively affect spatial awareness in the real world.	−0.19	3.76 ± 1.07	3.96 ± 1.02	−2.14	0.03 *	−0.19
Engaging in VGs that require remembering spatial details improves spatial memory in the real world.	−0.18	3.79 ± 1.01	3.97 ± 0.09	−2.09	0.04 *	−0.15
VGs that involve spatial thinking tasks enhance the ability to solve spatial problems in other areas of your life.	−0.16	3.89 ± 1.02	4.05 ± 0.97	−1.83	0.07	
Mastering navigation skills in VGs leads to improved navigation abilities in real-world situations.	−0.17	3.92 ± 1.02	4.09 ± 0.93	−2.11	0.04 *	−0.17
Increased SA in VGs translates to increased awareness in real-world spatial contexts.	−0.17	3.71 ± 1.03	3.88 ± 1.04	−1.91	0.06	
Engaging in VGs requires a high level of sustained attention to detail.	−0.12	3.79 ± 0.97	3.92 ± 1.03	−1.38	0.17	
Playing VGs has positively affected memory skills, such as remembering information or strategies within the game.	−0.10	3.86 ± 1.07	3.96 ± 1.03	−1.13	0.26	
The storyline and character development in VGs affect SA development.	−0.19	3.88 ± 1.02	4.07 ± 0.97	−2.19	0.03 *	−0.19

* Significant difference (*p* ≤ 0.05); T = independent *t*-test; M: mean; SD: standard deviation; VG: video game; d^t^: Cohen’s *d*.

**Table 5:** The relationship between video game exposure and their opinion about specific features, in-game activities, and visual cues of VGs that contribute most to developing spatial skills among the participants (*n* = 566).

Characteristics	Responses	Overall N. (%)	VG Exposure		$\chi^{2}$ Test	*p* Value
			Infrequent N. (%)	Frequent N. (%)		
Specific features in video games do you think contribute most to developing spatial skills?						
3D	No	235 (41.5)	80 (34.0)	155 (66.0)	2.59	0.72
	Yes	331 (58.5)	118 (35.6)	213 (64.4)		
Map exploration	No	193 (34.1)	66 (34.2)	127 (65.8)	1.08	0.85
	Yes	373 (65.9)	132 (35.4)	241 (64.6)		
Puzzle-solving	No	157 (27.7)	56 (35.7)	101 (64.3)	1.09	0.84
	Yes	409 (72.3)	142 (34.7)	267 (65.3)		
Virtual navigation	No	386 (68.4)	137 (35.5)	249 (64.5)	1.97	0.77
	Yes	179 (31.6)	61 (34.1)	118 (65.9)		
Dynamic terrain changes	No	373 (65.9)	132 (35.4)	241 (64.6)	1.08	0.85
	Yes	193 (34.1)	66 (34.2)	127 (65.8)		
In-game activities do you think require the most precise spatial awareness?						
Aiming	No	164 (29.0)	55 (33.5)	109 (66.5)	1.029	0.69
	Yes	402 (71.0)	143 (35.6)	259 (64.4)		
Jumping between platforms	No	390 (68.9)	134 (34.4)	256 (36.4)	0.89	0.74
	Yes	176 (31.1)	64 (36.4)	112 (63.6)		
Solving puzzles	No	374 (66.1)	125 (33.4)	249 (66.6)	1.21	0.70
	Yes	192 (33.9)	73 (38.0)	119 (62.0)		
Navigating mazes	No	313 (55.3)	111 (35.5)	202 (64.5)	1.02	0.86
	Yes	253 (44.7)	87 (34.4)	166 (65.6)		
Visual cues in video games that have the greatest impact on improving spatial skills?						
Shadows and lighting	No	224 (39.6	75 (33.5)	149 (66.5)	0.98	0.59
	Yes	342 (60.4)	123 (36.0)	219 (64.0)		
Depth of field	No	330 (58.3)	111 (33.6)	219 (66.4)	0.62	0.84
	Yes	236 (41.7)	87 (36.9)	149 (63.1)		
Perspective	No	162 (28.6)	47 (29.0)	115 (71.0)	3.99	0.05 *
	Yes	404 (71.4)	151 (37.4)	253 (62.6)		
Proximity indicators	No	360 (63.6)	125 (34.7)	235 (65.3)	0.029	0.93
	Yes	206 (36.4)	73 (35.4)	133 (64.6)		
Object scaling	No	351 (62.0)	124 (35.3)	227 (64.7)	0.058	0.86
	Yes	215 (38.0)	74 (34.4)	141 (65.6)		
Total		566 (100.0)	198 (35.0)	368 (65.0)		

* *p*-value is statistically significant ≤ 0.05; VG: video game.

## Data Availability

The datasets used and/or analyzed during the current study are available from the corresponding author on reasonable request. The data are not publicly available due to privacy and confidentiality considerations, as the dataset contains sensitive information collected from participants, which could potentially compromise their anonymity and personal information.
